# Amino Acid Composition, Antioxidant and Antihypertensive Activity in Extracts from Mexican Añejo Cheese

**DOI:** 10.17113/ftb.62.03.24.8533

**Published:** 2024-09

**Authors:** Verenice Torres-Salas, Blanca E. Hernández-Rodríguez, Javier Vioque-Peña, Julio Girón-Calle, Manuel Alaiz, Arturo Hernández-Montes, Holber Zuleta-Prada

**Affiliations:** 1 Posgrado en Ciencia y Tecnología Agroalimentaria, Departamento de Ingeniería Agroindustrial, Universidad Autónoma Chapingo, Km. 38.5 Carretera México-Texcoco, 56230 Chapingo, Estado de México, Mexico; 2 Departamento de Preparatoria Agrícola, Universidad Autónoma Chapingo, Km. 38.5 Carretera México-Texcoco, 56230 Chapingo, Estado de México, Mexico; 3 Instituto de la Grasa (C.S.I.C.), Universidad Pablo de Olavide, Cta de Utrera km. 1, 41089-Sevilla, Spain

**Keywords:** ripened añejo cheese, amino acid composition, antioxidant activity, antihypertensive activity

## Abstract

**Research background:**

Authentic Mexican cheeses have potential health benefits, although there are few studies on their bioactive components. In this study, we analysed soluble extracts from añejo cheese from Zacazonapan (Mexico), obtained from two dairies (A and B) that used milk from cows of different breeds and differed in processing.

**Experimental approach:**

The soluble extracts of Zacazonapan añejo cheese during ripening (0, 30, 95 and 180 days) were used to determine proximate composition, amino acid composition, peptide profile, molecular mass profile, antioxidant and antihypertensive activities.

**Results and conclusions:**

The molecular mass of the released protein fragments ranged from 0.10 to 22.43 kDa. During ripening, amino acids such as Pro, His, Tyr, Trp, Met, Cys, Ala, Gly, Leu and Val were present, which are associated with antioxidant activity. The inhibition of β-carotene bleaching was below 50 % at all ripening times. A significant difference between the cheese samples was observed only after 180 days. Amino acids associated with angiotensin-I converting enzyme (ACE-I) inhibition were found in the extracts (Trp, Phe, Tyr, Pro, Lys and Arg). The highest activity was observed in cheese ripened for 180 days (IC_50_ of cheese A and B was 0.38 and 0.42 mg/mL, respectively).

**Novelty and scientific contribution:**

These results suggest that Zacazonapan añejo cheese is a potential source of antioxidant and antihypertensive peptides, which are influenced by the dairy factor (milk source and production technique) and ripening time. This is relevant as there are no reports of these bioactivities in this type of cheese.

## INTRODUCTION

Around 50 types of genuine artisanal cheeses have been identified in Mexico, including Zacazonapan añejo cheese, which is produced in the southwest of the State of Mexico. It is made from raw milk from free-grazing cows, calf rennet and salt (Araró, Michoacán, Mexico) (*1*, *2*). Cheese has an economic, social and cultural importance at regional level (*3*). These artisanal cheeses form a potential system for the release of biologically active compounds due to their native microflora, the type of milk used and the different production techniques, although studies on their bioactive components are limited (*4*).

Proteolysis during cheese ripening releases peptides, amino acids, amines, organic acids, esters, aldehydes and sulfur compounds, which are products of casein hydrolysis by the protease and peptidase activity of starter lactic acid bacteria (SLAB) and non-starter lactic acid bacteria (NSLAB) (*5*, *6*). Some of the peptides may contribute to human health by exhibiting antihypertensive, antioxidant, antimicrobial, opioid, immunomodulatory, hypocholesterolemic and mineral-binding bioactivities (*7*, *8*). Peptides change dynamically during ripening because some parts of casein are less favoured by certain enzymes as substrates and some of the released peptides are rapidly degraded while other peptides accumulate (*9*, *10*).

Antioxidant peptides can prevent oxidation in food and *in vivo* in molecules such as proteins, lipids, carbohydrates and DNA (*11*). The production of such peptides has been reported in Mexican cheeses such as aged cow’s milk cheese Cotija, Fresco de cabra, Crema de Chiapas, Fresco and Cocido from Sonora (*12*-*14*).

On the other hand, the antihypertensive effect of peptides is related to the inhibition of the angiotensin-I converting enzyme (ACE-I), which is associated with an increase in arterial pressure (*15*). Several synthetic drugs are used to inhibit ACE-I activity, such as captopril (competitive inhibitor), but they are associated with side effects (*16*, *17*). Hence the interest in the search for natural sources of ACE-I inhibitors, such as biopeptides found in Mexican cheeses such as Cotija, fresh cow’s milk cheese made with *Lactococcus lactis* ssp. *lactis*, *Enterococcus faecium* and *Lactobacillus casei* and fresh goat’s milk cheese from Coatepec, Veracruz (*12*, *13*, *18*).

Due to the regional importance of añejo cheese from Zacazonapan and the lack of information on its proximal composition and biological activity, the aim of this research is to determine whether aqueous extracts of the cheese are a potential source of peptides with antioxidant and ACE-I inhibitory activities. We also considered the determination of response variables associated with peptide formation, such as amino acid content, peptide profile and molecular mass of peptides formed during cheese ripening.

## MATERIALS AND METHODS

### Materials and reagents

Analytical reagents d-, l-α-aminobutyric acid, diethyl ethoxymethylenemalonate, β-carotene, linoleic acid, Tween 20, butylated hydroxytoluene tetrahydrate (BHT), hippuric acid (HA), *N*-hippuryl-l-histidyl-l-leucine hydrate (HHL), angiotensin-I converting enzyme (ACE-I, E.C. 3.4.15.1, rabbit lung, 1U) and amino acid standard were purchased from Sigma Chemical Co., Merck (St. Louis, MO, USA). The molecular mass markers for fast protein liquid chromatography (FPLC) were blue dextran (2000 kDa), cytochrome C (12.5 kDa), aprotinin (6512 Da), bacitracin (1450 Da), cytidine (246 Da) and glycine (75 Da), which were purchased from Amersham Pharmacia LKB Biotechnology (Uppsala, Sweden).

### Cheese-making procedure and sampling

**Fig. S1:**
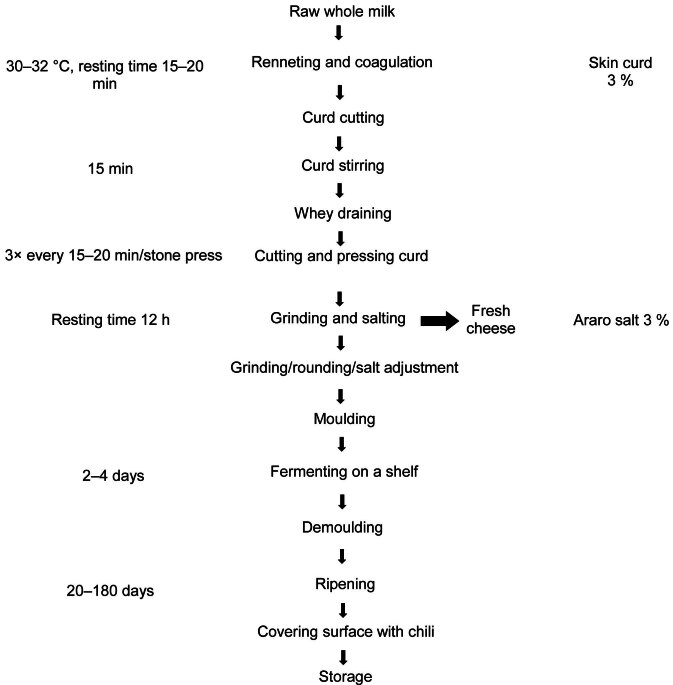
Flow chart for the production of Zacazonapan añejo cheese in dairy A

**Fig. S2:**
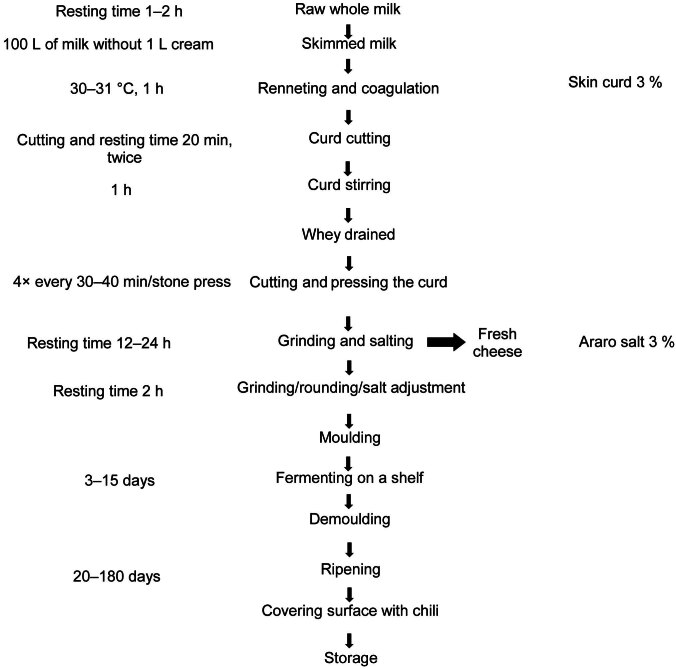
Flow chart for the production of Zacazonapan añejo cheese in dairy B

The cheese samples were obtained from two dairies (A and B) in Zacazonapan, State of Mexico, Mexico (N19°00'17" W100°12'55" and N19°16'17 W100°18'13", respectively). In each dairy, 1 kg of cheese was made from a batch of raw cow’s milk (without starter culture), calf rennet and 2–4 % salt (Araró, Michoacán, Mexico) of 90 % purity, curdled in the same vat and stored at 15 °C and 85 % relative humidity throughout the ripening period. Three samples of cheese from each dairy were analysed after 0, 30, 95 and 180 days of ripening. Cheese A was made from raw milk from free-grazing Brown Swiss cows, while cheese B was made using a standardised method and raw milk from free-grazing cows (crossbreed of Zebu and Brown Swiss) (Fig. S1 and Fig. S2).

### Extraction of water-soluble extracts

The water-soluble extracts were prepared according to Rae *et al.* (*19*) with minor modifications. A portion of cheese (250 g) was mixed with 750 mL of distilled water and homogenised for 5 min (Osterizer blender; John Oster Manufacturing Co, Racine, WI, USA). The resulting mixture was shaken in an orbital incubator (MaxQ SHKE4450 model 4333; Thermo Fisher Scientific, Waltham MA, USA) at 150 rpm and 40 °C for 1 h. The pH of the mixture was adjusted to 4.5 with 1 M HCl. The mixture was then centrifuged at 8801×*g* for 10 min at 4 °C (centrifuge model 5810; Eppendorf, Hamburg, Germany). The pellet and the upper fat layer were discarded, while the water-soluble extract was filtered by gravity through a cotton layer and then through filter paper No. 42. The extracts were stored at −20 °C and lyophilised at a pressure 1.5 Pa and a temperature of -50 °C for 48 h using a 2.5 Free Zone device (Labconco Corporation, Kansas, MO, USA).

### Determination of proximal composition of water-soluble extracts

Moisture content was determined by weighing 250 mg of the sample and drying it at 70 °C for 24 h. The samples were then calcined at 550 °C for 24 h to determine the ash mass fraction (*20*). The protein content (*21*), on the other hand, was calculated by subtracting the free amino acid content from the total amino acid content determined by the methods mentioned in the following sections.

The method described by Folch *et al.* (*22*) was used for the determination of lipid content. A mass of 50 mg of sample was weighed into a 1-mL vial and mixed with 500 µL of a chloroform/methanol solution (2:1) and then shaken at 300 rpm (Stuart SSM1 orbital shaker; Bibby Sterilin Ltd., Stone, UK) for 20 min at 25 °C. The samples were then centrifuged at 370×*g* (centrifuge model 5415R; Eppendorf) for 15 min at 25 °C, the supernatant was collected and the pellet was washed with 500 µL of the chloroform/methanol mixture (2:1). The obtained supernatants were filled up to 1 mL with a chloroform/methanol mixture (2:1), washed with 200 µL of 0.9 % NaCl (*m*/*V*) and centrifuged at 370×*g* for 4 min (centrifuge model 5415R; Eppendorf). The upper phase was discarded and the lower phase was washed with 200 µL of distilled water. The tube was then centrifuged at 370×*g* for 4 min (model 5415R; Eppendorf), the upper phase was discarded and the lower phase was concentrated with nitrogen. Finally, the tube was weighed and the lipid content was determined by mass difference.

Reducing sugars were determined as described by Dubois *et al.* (*23*). Briefly, 8 mg of sample were mixed with 2 mL of distilled water and shaken at 300 rpm for 1 h (Stuart SSM1 orbital shaker; Bibby Sterilin Ltd), then centrifuged at 12 433×*g* for 4 min at 4 °C (model 5415R; Eppendorf). Then, 1 mL of 5 % (*m/V*) phenol and 5 mL of concentrated H_2_SO_4_ were added, the samples were stirred and allowed to rest for 20 min. The absorbance was measured at 480 nm (Multiskan model 1500; Thermo Lab Systems, Vantaa, Finland). A standard glucose curve (5–100 µg) was constructed. Proximal composition analysis was done in triplicate and results were expressed in g/100 g lyophilised extract.

### Determination of total amino acid profile of water-soluble extracts

The amino acid composition of the soluble extracts was determined according to Alaiz *et al.* (*21*), except for tryptophan, which was analysed as described by Yust *et al.* (*24*). Amino acid profiles were determined in a high-performance liquid chromatograph (HPLC; Beckman-Coulter, Brea, CA, USA) with a separation module 126, a rheodyne valve-type injector with 20 µL loop, a model 166 programmable UV/Vis detector module and a reversed phase column (300 mm×3.9 mm, 4-μm, Novapack C18; Waters, Milford, MA, USA). The determination was carried out at 18 °C, a flow rate of 0.9 mL/min, with a gradient formed by solution A (25 mM sodium acetate and 0.02 % (*m/V*) sodium azide at pH=6) and acetonitrile as solution B. The gradient conditions were 0-3 min, linear gradient from A/B (91:9) to A/B (86:14); 3-13 min elution with A/B (86:14); 13-30 min linear gradient from A/B (86:14) to A/B (69:31); 30-35 min elution with A/B (69:31). Data acquisition and processing was performed with the 32 Karat v. 7.0 (*25*).

### Determination of total free amino acids of water-soluble extracts

The total free amino acids were determined according to Megías *et al.* (*26*). Amino acid derivatisation was performed with diethyl ethoxymethylenemalonate, using aminobutyric acid as internal standard (*21*). Then 20 µL of the supernatant were injected into an HPLC device with the characteristics described above. The gradient conditions and data analysis were the same as those used for the determination of the total amino acid profiles.

### Determination of peptide profiles of water-soluble extracts

Peptide profiles were determined using a molecular separation method based on hydrophobicity differences (*27*). The soluble extracts were injected into a previously described HPLC (Beckman-Coulter) using a Discovery BIO Wide Pore C18 reverse column, 25 cm×4.6 mm, 5 μm (Supelco, Bellefonte, PA, USA). The solvent used was A: water with 0.1 % (*V*/*V*) trifluoroacetic acid and B: acetonitrile with 0.1 % (*V*/*V*) trifluoroacetic acid. A linear gradient from A/B (100:0) to A/B (70:30) was programmed for 60 min at a flow rate of 1 mL/min. The absorbance was measured at 215 nm and the column temperature was kept at 25 °C. Data were recorded and processed using 32 Karat v. 7.0 (*25*).

### Determination of the molecular mass profile of the peptides in the water-soluble extracts

The molecular mass profiles of soluble extracts of the cheese were determined according to Hernández-Jabalera *et al.* (*27*) by injecting 500 µL of sample at a concentration of 20 mg/mL through a gel filtration chromatograph on a Superdex 10/300 GE GL column (Healthcare, Uppsala, Sweden) coupled to an FPLC AKTA purifier system equipped with a P-900 binary pump, a UPC-900 UV detector (GE Healthcare, Piscataway, NJ, USA). A 0.75 M ammonium bicarbonate buffer with a flow rate of 0.5 mL/min was used as the mobile phase. The absorbance of the elution was recorded at 215 nm.

### Determination of the antioxidant activity of soluble extracts of Zacazonapan añejo cheese by β-carotene bleaching assay

Antioxidant activity was determined using β-carotene bleaching assay as described by Hernández-Jabalera *et al.* (*27*), with minor modifications. Briefly, 1 mL of the β-carotene solution (2 mg of β-carotene dissolved in 1 mL of chloroform) was mixed with 20 µL of linoleic acid and 200 µL of Tween 20 and flushed with nitrogen to remove chloroform. The rinsed β-carotene was mixed with 25 mL of oxygen-saturated distilled water to obtain a translucent mixture (absorbance <1 at 450 nm). A volume of 2 µL of soluble cheese extract (12 μg of protein per μL) and 200 μL of translucent mixture were stirred and incubated at 50 °C in the dark. The absorbance (450 nm) was measured at 10-minute intervals for 1 h (Multiskan model 1500, Thermo Lab Systems). The control was prepared by replacing the sample with distilled water, while in the positive control the sample was replaced with BHT (12 μg/μL).

Three different variables were used to calculate the antioxidant activity, firstly the degradation rate (DR) using the following equation (*28*):


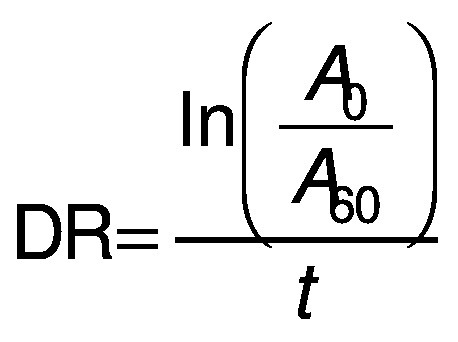
 /1/

where *A*_0_ and *A*_60_ are the absorbances at *t*=0 and 60 min, respectively.

The second variable, the percentage of β-carotene bleaching inhibition (in %), relative to a control, was calculated according to the following equation (*28*):


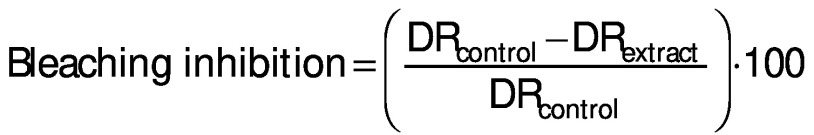
 /2/

where DR_control_ and DR_extract_ are the degradation rates of β-carotene in the absence and in the presence of the extract, respectively.

Finally, the coefficient of antioxidant activity (CAA) was calculated according to Mallet *et al*. (*29*), using the following equation:


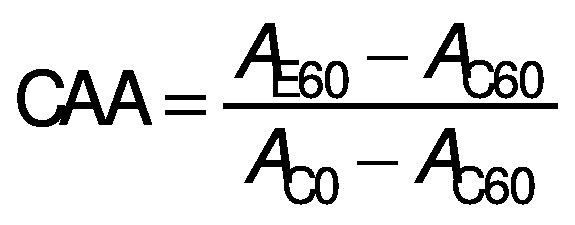
 /3/

where *A*_E60_ and *A*_C60_ are the absorbance values at 450 nm after 60 min of the antioxidant and the control mixture, respectively, and *A*_C0_ is the absorbance of the control at *t*=0 min.

### Determination of the ACE-I inhibitory activity of soluble extracts

The ACE-I inhibitory activity was determined according to Wanasundara *et al.* (*30*). Soluble extracts were centrifuged (model 5810; Eppendorf) at 5000×*g* for 10 min at 4 °C, and the supernatants were recovered and adjusted to pH=8.3. A volume of 100 µL of each extract (0.3 mg protein per mL) was mixed with 200 µL of 5 mM HHL in 0.1 M borate buffer (pH=8.3), 0.3 M NaCl and 100 µL ACE-I (2 mU). The mixture was incubated at 37 °C for 60 min and the reaction was stopped by adding 300 µL of 1 M HCl. Then, the hippuric acid (HA) in the mixture was quantified with a reversed phase high-performance liquid chromatography (RP-HPLC, Series 1260; Agilent^®^, Santa Clara, CA, USA) using a C18 column (250 mm×4.6 mm, 5 μm, 300 Å; Jupiter^®^, Phenomenex, Torrance, CA, USA). The analysis conditions were as follows: injection volume of 20 µL, mobile phase 12.5 % (*V*/*V*) acetonitrile in water at pH=3, flow rate 1 mL/min and column temperature at 25 °C. In the control, the extract was replaced by deionised water. ACE-I inhibition (in %) by the soluble extracts was determined using the following equation:


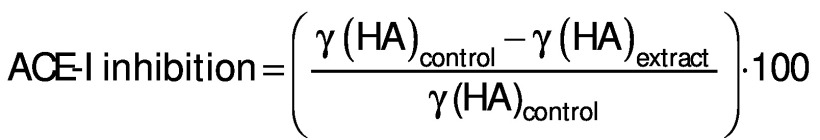
 /4/

where *γ*(HA)_control_ is the hippuric acid concentration in the control (with enzyme and without extract, mg/L), and *γ*(HA)_extract_ is the hippuric acid concentration in the assay (with enzyme and with extract, mg/L). In addition, the IC_50_ value for extracts after 180 days was determined by regression analysis of ACE-I inhibition (in %) *versus* protein concentration (0.1, 0.2, 0.3, 0.4 and 0.5 mg/mL).

### Statistical analysis

In this study, a completely randomised design with a factorial arrangement was used, where the first factor was dairies with two levels (A and B) and the second factor was ripening time with four levels (0, 30, 95 and 180 days). Mean values were compared using the least significant difference (LSD) method with α=0.05 using the SAS v. 9.0 statistical program (*31*).

## RESULTS AND DISCUSSION

### Proximal composition of the soluble extracts of Zacazonapan añejo cheese

Moisture was significantly different (p≤0.05) in relation to the dairy factor, 1.85 and 2.85 g/100 g of freeze-dried extract of cheeses A and B, respectively. Regarding protein content, lipids, reducing sugars and ash as response variables (Table 1), there was a significant difference (p≤0.05) in the interaction between the dairy and ripening time factors. The protein mass fraction decreased, which could be related to proteolysis by residual rennet, milk enzymes, starter culture enzymes and secondary microbiota, which contribute to the formation of polypeptides, oligopeptides, free amino acids, amines, organic acids, esters, aldehydes and sulfur compounds from caseins (*32*). The produced compounds can be consumed by the microorganisms present in the cheese, because their growth depends on them (*33*).

**Table 1:** Mass fractions of proteins, lipids, reducing sugars and ash in the soluble extracts of añejo cheese from Zacazonapan, Mexico

		Dairy		
Compound	*t*(ripening)/day	A	B	
		*w*(compound)/(g/100 g)		
Protein	0	(29.4±0.5)^ax^		(30.5±0.5)^ax^
	30	(27.8±0.5)^abx^		(24.8±0.5)^by^
	95	(28.3±0.5)^ax^		(23.5±0.5)^by^
	180	(26.4±0.5)^bx^		(14.7±0.5)^cy^
Lipid	0	(6.5±0.3)^by^		(8.3±0.3)^bx^
	30	(8.3±0.3)^ay^		(10.2±0.3)^ax^
	95	(8.1±0.2)^ax^		(8.8±0.2)^bx^
	180	(8.7±0.2)^ay^		(10.9±0.3)^ax^
Reducing sugar	0	(2.5±0.4)^ay^		(7.8±0.4)^ax^
	30	(0.4±0.4)^bx^		(0.6±0.4) ^bx^
	95	(0.4±0.3)^bx^		(0.5±0.4)^bx^
	180	(0.5±0.4)^bx^		(0.6±0.4)^bx^
Ash	0	(58.7±0.7)^bcx^		(5.0±0.7)^by^
	30	(59.8±0.6)^bx^		(59.2±0.7)^ax^
	95	(57.6±0.7)^cx^		(54.9±0.6)^by^
	180	(66.0±0.7)^ax^		(59.2±0.7)^ay^

Mean value±standard error of three independent determinations expressed on freeze-dried extract mass basis. Different letters in a column (a, b and c) or in a row (x and y) indicate statistical differences between the ripening time and dairy factors (p≤0.05), respectively

On the other hand, the lipid mass fraction may be related to lipolytic enzymes from lactic acid bacteria (LAB), which produce free fatty acids, esters, tri-, di- and monoacylglycerides (*34*). The decrease in reducing sugar mass fraction may be related to LAB metabolism of residual lactose to lactate (*5*).

The ash mass fraction was higher than in other cheeses such as Parmigiano Reggiano (13.8 %) and Grana Padano (13.6 %) salted in brine (*35*). The high mass fraction of inorganic matter in Zacazonapan cheese extracts is related to the salt used in its production (*2*).

### Total amino acid profile of the soluble extracts of Zacazonapan añejo cheese

The mass fraction of the amino acids Asp+Asn, His and Lys presented significant differences between the extracts of cheese A and B, namely 2.21, 2.14 and 2.70 for cheese A and 2.06, 1.97 and 2.49 g/100 g of freeze-dried extract for cheese B, respectively (data not shown). In addition, a statistically significant difference (p≤0.05) was found for Asp+Asn, His, Pro and Lys in relation to ripening time. In general, the highest mass fraction of these amino acids was reached at 95 days of ripening, with values of 2.34, 2.27, 2.72 and 2.92 g/100 g freeze-dried extract, respectively. This content decreased after 180 days and showed similar values after 30 days of ripening (data not shown). The observed differences between the soluble extracts and the ripening time are due to factors related to the manufacturing process, temperature conditions, type of coagulant (calf rennet, plant proteases, *etc*.), starter cultures, and ripening conditions (time, temperature and humidity) (*17*, *36*). In this case, in addition to other variants, there are also differences in the fermentation time, which is longer for cheese B (3–15 days) than for cheese A (2–4 days) (Fig. S1 and Fig. S2 respectively).

Table 2 shows the interaction of the dairy and ripening time factors, significant differences (p≤0.05) were observed for amino acids such as Tyr, Trp, Met, Cys, Ala, Gly, Leu and Val, which are important in peptides with antioxidant activity. In addition, amino acids associated with the inhibitory activity of ACE-I such as Trp, Phe, Tyr and Arg were present. The highest amino acid mass fraction was Glu+Gln due to the abundance of Glu in the caseins (*37*), and the lowest was Cys.

**Table 2:** Mass fractions of total amino acids in añejo cheese soluble extracts with significant difference (p≤0.05) of the interaction of dairy and ripening time factors

		*t*(ripening)/day			
Amino acid	Dairy	0	30	95	180
		*w*(amino acid)/(g/100 g)			
Glu+Gln	A	(5.6±0.3)^bx^	(6.9±0.3)^ax^	(7.2±0.3)^ax^	(6.8±0.3)^ax^
	B	(6.2±0.3)^ax^	(4.4±0.3)^cy^	(5.7±0.0)^aby^	(4.6±0.3)^bcy^
Ser	A	(1.45±0.06)^by^	(1.52±0.06)^bx^	(2.07±0.05)^ax^	(2.01±0.06)^ax^
	B	(1.69±0.06)^ax^	(1.10±0.06)^cy^	(1.52±0.06)^aby^	(1.35±0.06)^by^
Gly	A	(0.55±0.03)^bx^	(0.69±0.03)^bx^	(0.89±0.04)^ax^	(0.84±0.04)^ax^
	B	(0.54±0.04)^acx^	(0.49±0.03)^bcy^	(0.62±0.03)^ay^	(0.57±0.04)^aby^
Thr	A	(1.11±0.02)^by^	(0.94±0.02)^cx^	(1.29±0.02)^ax^	(1.29±0.02)^ax^
	B	(1.24±0.02)^ax^	(0.69±0.02)^dy^	(0.93±0.02)^by^	(0.78±0.02)^cy^
Ile	A	(1.42±0.08)^cx^	(1.82±0.08)^bx^	(2.44±0.08)^ax^	(2.55±0.08)^ax^
	B	(1.53±0.08)^ax^	(1.27±0.08)^by^	(1.68±0.08)^ay^	(1.50±0.08)^aby^
Arg	A	(1.03±0.03)^ax^	(1.00±0.02)^ax^	(0.99±0.03)^ax^	(0.86±0.03)^bx^
	B	(0.92±0.03)^ay^	(0.71±0.03)^by^	(0.55±0.02)^cy^	(0.41±0.03)^dy^
Ala	A	(0.72±0.04)^cx^	(0.90±0.05)^bx^	(0.97±0.05)^bx^	(1.16±0.05)^ax^
	B	(0.71±0.05)^ax^	(0.52±0.04)^by^	(0.67±0.04)^ay^	(0.58±0.04)^aby^
Tyr	A	(0.48±0.03)^ay^	(0.46±0.02)^ax^	(0.48±0.02)^ax^	(0.42±0.03)^ax^
	B	(0.65±0.03)^ax^	(0.29±0.02)^by^	(0.29±0.02)^by^	(0.18±0.03)^cy^
Val	A	(1.91±0.06)^by^	(1.93±0.06)^bx^	(2.78±0.06)^ax^	(2.65±0.06)^ax^
	B	(2.21±0.06)^ax^	(1.32±0.06)^dy^	(1.81±0.06)^by^	(1.53±0.06)^cy^
Met	A	(0.51±0.05)^bx^	(0.47±0.05)^bx^	(0.86±0.05)^ax^	(0.82±0.05) ^ax^
	B	(0.48±0.05)^abx^	(0.38±0.05)^acx^	(0.53±0.05)^ay^	(0.3±0.05)^bcy^
Cys	A	(0.024±0.002)^ay^	(0.024±0.002)^ax^	(0.025±0.002)^ax^	(0.027±0.002)^ax^
	B	(0.041±0.002)^ax^	(0.007±0.002)^cy^	(0.016±0.002)^by^	(0.004±0.002)^cy^
Trp	A	(0.85±0.02)^ax^	(0.78±0.02)^acx^	(0.79±0.02)^abx^	(0.76±0.02)^bcx^
	B	(0.68±0.02)^by^	(0.80±0.02)^ax^	(0.78 ±0.02)^ax^	(0.77±0.02)^ax^
Leu	A	(2.91±0.01)^cx^	(3.3±0.01)^bx^	(4.18±0.01)^ax^	(3.98±0.01)^ax^
	B	(3.15±0.01)^ax^	(2.7±0.01)^bcy^	(2.80±0.01)^aby^	(2.74±0.01)^acy^
Phe	A	(1.47±0.06)^cx^	(1.69±0.06)^bx^	(2.01±0.06)^ax^	(1.81±0.06)^bx^
	B	(1.65±0.06)^ax^	(1.06±0.06)^cy^	(1.38±0.06)^by^	(1.14±0.06)^cy^

Mean value±standard error of three independent determinations expressed on freeze-dried extract mass basis. Different letters in a row (a, b, c and d) or in a column (x and y) indicate a significant difference (p≤0.05) between the ripening time and the dairy factors, respectively

On the other hand, the Arg mass fraction in the extract of cheese A at *t*=0 day (1.03 g/100 g freeze-dried extract) showed a difference of 0.17 g compared to *t*=180 day (0.86 g/100 g freeze-dried extract), while the difference for the same time interval was greater for the extracts of cheese B (0.51 g), namely 0.41 g/100 g freeze-dried extract at *t*=180 day and 0.92 g/100 g freeze-dried extract on *t*=0 day. The use of Arg by some bacteria as an energy source has been associated with its decrease during cheese ripening (*9*). Branched-chain amino acids are susceptible to decarboxylation reactions, while aromatic amino acids are susceptible to deamination reactions, producing volatile organic compounds such as amines, esters and aldehydes (*5*).

### Free amino acid profile of the soluble extracts of Zacazonapan añejo cheese

The total free amino acid mass fraction of the soluble extracts showed a significant difference (p≤0.05) for the interaction of the dairy and ripening time factors (Table 3). The highest mass fraction of total free amino acids was obtained after 180 days (8.0 and 12.6 g/100 g freeze-dried extract of cheeses A and B, respectively). Ayag *et al.* (*38*) reported similar findings in Turkish cheeses ripened between one and nine months: Tulum (0.53–0.14 g/100 g), Kasar (0.10–0.94 g/100 g) and the lowest mass fraction was reported for Beyaz Peynir (0.27–0.29 g/100 g). This increase is due to the hydrolysis of milk proteins (mainly caseins) by bacterial proteases during prolonged ripening, resulting in low-molecular-mass protein fragments, peptides and finally free amino acids (*38*, *39*). Salt content reduces water availability, which leads to the formation of protein aggregates that limit the access of proteases to peptide bonds and thus reduce protein hydrolysis (*37*).

**Table 3:** Mass fractions of total free amino acids in añejo cheese soluble extracts after ripening

	*t*(ripening)/day			
Dairy	0	30	95	180
	*w*(total amino acid)/(g/100 g)			
A	(0.4±0.4)^dx^	(3.5±0.4)^cy^	(5.6±0.4)^by^	(8.0±0.4)^ay^
B	(0.5±0.4)^dx^	(4.9±0.4)^cx^	(9.5±0.4)^bx^	(12.6±0.4)^ax^

Mean value±standard error of three independent determinations expressed on freeze-dried extract mass basis. Different letters in a row (a, b, c and d) or in a column (x and y) indicate significant differences between the ripening times (p≤0.05) and the dairy factor, respectively (p≤0.05)

Statistical analysis indicated significant difference (p≤0.05) in the mass fraction of the amino acids Thr, Leu and Asp+Asn in relation to the dairy factor, with cheese A standing out with a mass fraction of 1.2 and 0.4 g/100 g of freeze-dried extract for Leu and Asp+Asn, respectively (data not shown). In addition, an increase in the content of Asp, Gly, Thr, Tyr, Met, Leu and Phe was found during the ripening of Zacazonapan añejo cheese (data not shown). These results differ from those found by Ardö *et al.* (*40*) in Herrgård, Samso and Dambo cheeses, who reported an increase in free amino acids such as Pro, Lys, Gly and Ile and a decrease in Leu, Phe, Gln and Asn during cheese ripening.

The free amino acids with the highest mass fraction after 180 days were Glu, Ser, Ala, Pro, Val, Ile and Lys in both cheeses (Table 4). Cheese B had higher mass fraction of Glu, Asn, Ser, Gln, His, Arg, Pro, Ile and Lys than cheese A at the end of ripening. Researchers reported an increase in specific amino acids during the ripening of some cheeses such as Beyaz Peynir (His, Ile and Lys), Kasar (Glu, His, Tyr, Ile, Phe and Trp), Tulum (His, Arg, Tyr, Ile, Leu and Trp) and Teleme (Leu, Glu, Phe, Val and Lys) (*38*, *41*). These results show that the free amino acid profile is characteristic of each cheese as a result of the enzymatic system present in the raw milk and later on in the cheese matrix, as well as the interconversion and degradation of amino acids during ripening (*36*). The presence of amino acids such as Val, Phe and Leu is related to the proteolysis of α_s1_ casein, which is rich in these amino acids and whose degradation occurs during the first days of ripening (*41*). The aminotransferase activity on the amino acid Gln or Asn produces amino groups that are transferred to α-ketoglutarate, oxaloacetate and pyruvate yielding Glu, Asp and Ala, respectively (*40*).

**Table 4:** Mass fractions of free amino acids in añejo cheese soluble extracts with significant difference (p≤0.05) of the interaction between dairy and ripening time factors

		*t*(ripening)/day			
Amino acid	Dairy	0	30	95	180
		*w*(free amino acid)/(g/100 g)			
Glu	A	(0.03±0.04)^cx^	(0.34±0.04)^ax^	(0.19±0.04)^by^	(0.24±0.04)^aby^
	B	(0.14±0.04)^dx^	(0.32±0.03)^cx^	(1.11±0.04)^bx^	(1.62±0.04)^ax^
Asn	A	(0.002±0.007)^ax^	(0.009±0.006)^ay^	(0.015±0.007)^ay^	(0.013±0.007)^ay^
	B	(0.007±0.007)^cx^	(0.129±0.007)^bx^	(0.220±0.007)^ax^	(0.242±0.007)^ax^
Ser	A	(0.0003±0.007)^bcx^	(0.024±0.007)^ay^	(0.008±0.007)^acy^	(0.018±0.007)^aby^
	B	(0.001±0.005)^dx^	(0.061±0.007)^cx^	(0.249±0.007)^bx^	(0.423±0.007)^ax^
Gln	A	(0.000±0.009)^ax^	(0.01±0.01)^ay^	(0.003±0.01)^ay^	(0.02±0.01)^ay^
	B	(0.00±0.01)^dx^	(0.10±0.01)^cx^	(0.24±0.01)^ax^	(0.17±0.01)^bx^
His	A	(0.02±0.02)^ax^	(0.05±0.02)^ay^	(0.02±0.02)^ay^	(0.07±0.02)^ay^
	B	(0.02±0.02)^dx^	(0.16±0.02)^cx^	(0.50±0.02)^bx^	(0.62±0.02)^ax^
Arg	A	(0.000±0.003)^ax^	(0.000±0.003)^ax^	(0.003±0.003)^ay^	(0.009±0.003)^ay^
	B	(0.001±0.003)^cx^	(0.009±0.003)^cx^	(0.034±0.003)^bx^	(0.059±0.003)^ax^
Ala	A	(0.01±0.02)^dx^	(0.13±0.02)^cx^	(0.33±0.02)^bx^	(0.49±0.02)^ax^
	B	(0.01±0.01)^dx^	(0.11±0.02)^cx^	(0.26±0.01)^by^	(0.31±0.01)^ay^
Pro	A	(0.12±0.02)^bx^	(0.20±0.02)^ay^	(0.21±0.02)^ay^	(0.15±0.02)^aby^
	B	(0.08±0.02)^cx^	(0.34±0.02)^bx^	(0.82±0.02)^ax^	(0.76±0.02)^ax^
Val	A	(0.02±0.04)^dx^	(0.46±0.04)^cx^	(0.75±0.04)^bx^	(1.15±0.04)^ax^
	B	(0.03±0.04)^dx^	(0.35±0.03)^cx^	(0.74±0.03)^bx^	(0.90±0.03)^ay^
Cys	A	(0.0000±0.0003)^cx^	(0.0033±0.0003)^bx^	(0.0048 ±0.0003)^ax^	(0.0054±0.0003)^ax^
	B	(0.0000±0.0003)^bx^	(0.0033±0.0003)^ax^	(0.0000±0.0003)^by^	(0.0000±0.0003)^by^
Ile	A	(0.01±0.04)^dx^	(0.17±0.04)^cx^	(0.36±0.04)^by^	(0.61±0.04)^ay^
	B	(0.01±0.04)^dx^	(0.22±0.03)^cx^	(0.59±0.03)^bx^	(0.79±0.03)^ax^
Lys	A	(0.01±0.04)^bx^	(0.17±0.04)^ay^	(0.23±0.04)^ay^	(0.29±0.04)^ay^
	B	(0.05±0.04)^cx^	(0.53±0.04)^bx^	(1.44±0.04)^ax^	(1.54±0.04)^ax^

Mean value±standard error of three independent determinations expressed on freeze-dried extract mass basis. Different letters in a row (a, b, c and d) or column (x and y) indicate significant differences (p≤0.05) between the ripening time and the dairy factor, respectively

### Peptide profile of the soluble extracts of Zacazonapan añejo cheese

**Fig. 1:**
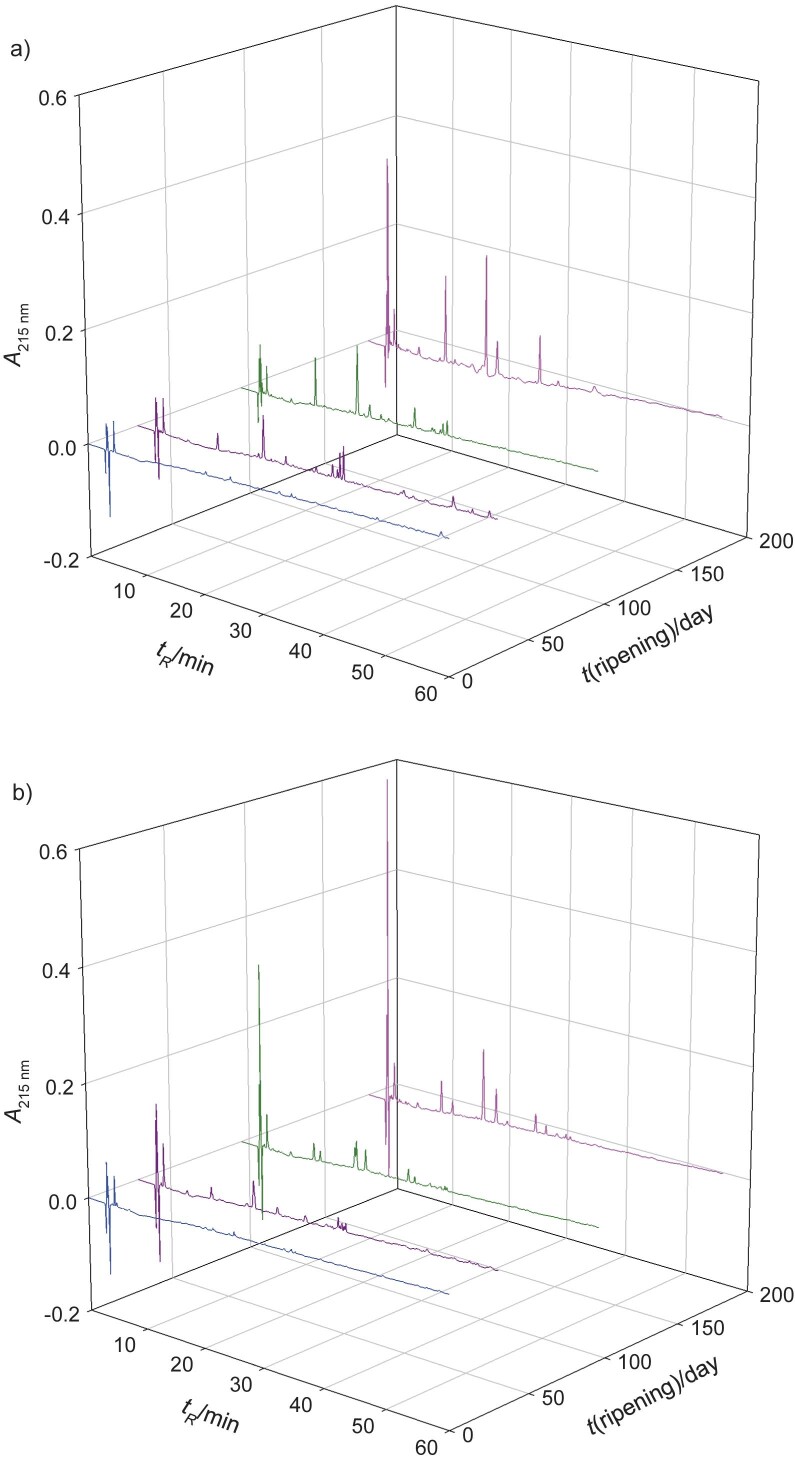
RP-HPLC peptide profiles of añejo cheese soluble extracts from: a) dairy A and b) dairy B at *t*(ripening times)=0 (blue), 30 (purple), 95 (green) and 180 (pink) days

The results show that the peptide profile changes depending on the ripening time, with a more complex peptide profile observed in cheese A than in cheese B after 180 days of ripening (Fig. 1). The maximum increase in peptide content in Turkish Tulum cheese was reported by Öztürk and Akın (*10*) at 180 days of ripening, especially due to the activity of aminopeptidase and carboxypeptidase. Hernández-Galán *et al.* (*13*) reported that the factors involved in the cheese processing and ripening produce a unique peptide profile for each type of cheese, including the breed of cattle, the resting time of the milk before curdling, the incubation time of the rennet in the milk and the curd cutting time (*42*). In this case, the dairies used cow’s milk of different breeds and the curd of cheese B was cut more frequently and at longer intervals than the curd of cheese A (Fig. S1 and Fig. S2). The resting time of cheese B was also longer than that of cheese A, which could lead to a higher amount of proteins and lipids and reduce the availability of water for the development of microorganisms (*43*). In particular, the quantity and diversity of bioactive peptides are the result of the microbiota living in the cheese ecosystems. The LAB influence the spatial diversification and temporal distribution of bioactive peptides through an equilibrium between their production and consumption (*7*).

### Native protein and peptide molecular mass profile of the soluble extracts of Zacazonapan añejo cheese

**Fig. 2:**
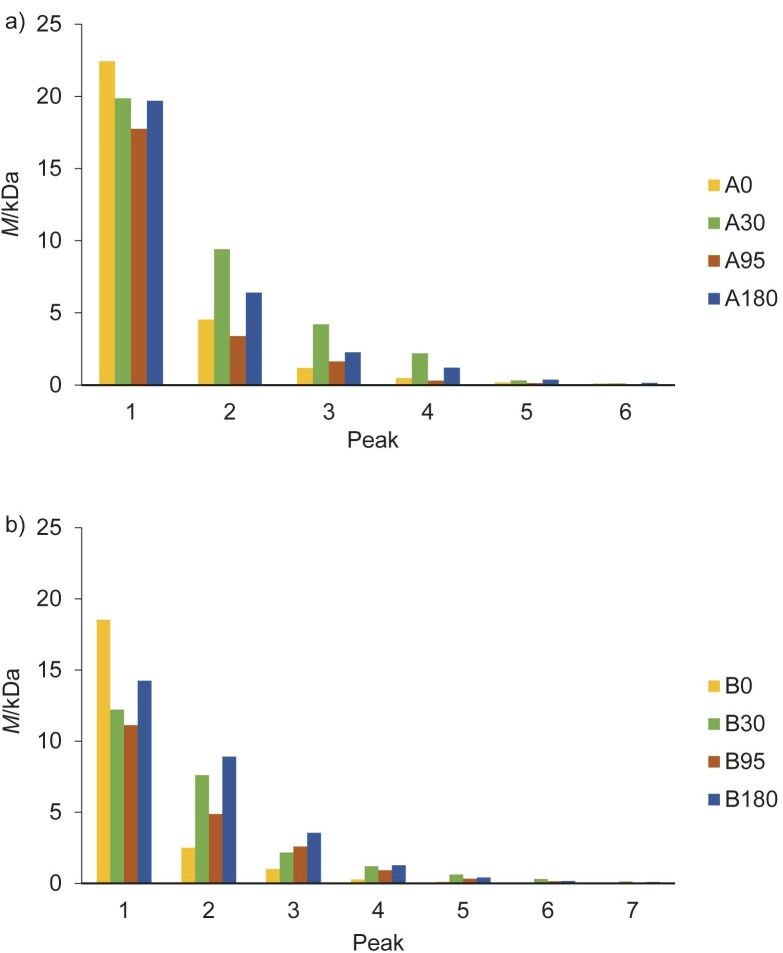
Molecular mass of proteins and peptides in añejo cheese soluble extracts from: a) dairy A and b) dairy B during *t*(ripening)=0, 30, 95 and 180 days

The molecular mass profiles determined for cheeses A and B at different ripening times are shown in Fig. 2. The peaks corresponding to the native proteins and peptides present in cheeses A and B were observed in the range of 0.12–22.43 kDa and 0.10–15.55 kDa, respectively. An increase in the content of peptides with small molecular mass was observed with increasing ripening time, which was related to the progression of the proteolysis reaction in the cheese. Several researchers have shown interest in peptide fractions smaller than 3 kDa in Mexican cheeses (cream from Chiapas, fresh and cooked cheese from Sinaloa) (*14*, *18*) and in fractions between 5 and 10 kDa in Cheddar cheese (*44*); all these fractions are associated with antioxidant and angiotensin-I converting enzyme (ACE-I) inhibitory activity, and some with antimicrobial activity.

### Antioxidant activity of the soluble extracts of Zacazonapan añejo cheese

The peptides with antioxidant activity are released during the production and ripening of cheese (*13*). For the interaction between the factors dairy and ripening time, the statistical analysis showed a significant difference (p≤0.05) for the variables β-carotene degradation rate (DR), antioxidant activity (AA) and coefficient of antioxidant activity (CAA). Table 5 shows that for cheese A the lowest DR was obtained at the beginning and at the end of the ripening period, while for cheese B the lowest DR was obtained at the beginning. The values obtained are higher than those of BHT (0.00127). The AA results for cheese A are consistent with the DR data, with the highest values obtained on day 0 ((45.3±1.9) %) and after 180 days (44.6±1.5) %), while for cheese B the highest AA value was obtained at the beginning of ripening ((44.7±1.5) %), both values being lower than the 92.4 % reported for BHT. Similarly, the highest CAA values for cheeses A and B were found on day 0 ((322.3±9.6) and (291.1±9.6), respectively) and after 180 days ((346.3±9.6) and (296.1±9.6), respectively) of ripening. In particular, most food-derived antioxidants have a molecular mass in the range of 500 to 1500 Da and sometimes contain hydrophobic amino acid residues such as Val or Leu at the N-terminal end of peptides, and Pro, His, Tyr, Trp, Phe, Gly, Met and Cys in their sequences (*45*, *46*). The results observed in Table 5 could be related to the presence of each of these amino acids in the Zacazonapan añejo cheese extracts.

**Table 5:** Degradation rate (DR), antioxidant activity (AA) and coefficient of antioxidant activity (CAA) in añejo cheese soluble extracts with significant difference (p≤0.05) of the interaction of dairy factor and ripening time

			*t*(ripening)/day			
Variable	BHT	Dairy	0	30	95	180
DR	(0.00127±0.00007)	A	(0.0097±0.0002)^bx^	(0.0106±0.0002)^ax^	(0.0099±0.0002)^abx^	(0.0096±0.0002)^by^
		B	(0.0090±0.0002)^bx^	(0.0104±0.0002)^ax^	(0.0105±0.0002)^ax^	(0.0110±0.0002)^ax^
AA/%	(92.4±0.4)	A	(45.3±1.9)^ax^	(11.4±1.9)^cy^	(37.3±1.9)^bx^	(44.6±1.5)^ax^
		B	(44.7±1.5)^ax^	(23.53±1.9)^cx^	(33.4±1.9)^bx^	(37.7±1.9)^by^
CAA	(881.2±5.6)	A	(322.3±9.6)^ax^	(71.7±9.6)^cy^	(268.0±9.6)^bx^	(346.3±9.6)^ax^
		B	(291.1±9.6)^ay^	(221.0±9.6)^bx^	(248.8±9.6)^bx^	(296.1±9.6)^ay^

Mean value±standard error of three independent determinations expressed on freeze-dried extract mass basis. Different letters in a row (a, b and c) indicate significant differences between the ripening times (p≤0.05) or different letters in a column (x and y) indicate significant differences between dairy factors (p≤0.05). BHT=butylated hydroxytoluene tetrahydrate

### ACE-I inhibitory activity of the soluble extracts of Zacazonapan añejo cheese

The soluble cheese extracts showed ACE-I inhibitory activity. Statistical analysis presented significant differences (p≤0.05) for the dairy and ripening time factors, but not for the interaction between them (p≥0.05). As for the dairy factor, the soluble extracts of cheese A inhibited ACE-I by (17.6±1.4) %, while the soluble extracts of cheese B inhibited it by (28.5±1.4) % (data not shown). Other authors have reported a 100 % inhibitory activity in different Mexican cheeses, such as Cotija, fresh goat’s milk cheese and fresh cow’s milk cheese (*12*, *13*, *18*).

Regarding the ripening time factor, the ACE-I inhibition increased from 6.5 to 39.3 % from day 0 to day 180, respectively (Table 6). This result is in contrast to that obtained by Taha *et al.* (*47*) for Domiati cheese, where a longer ripening time reduced the ACE-I inhibitory activity. It should be noted that ripening is influenced by different conditions such as heat treatment of milk, addition of starter cultures, temperature, duration of processing and type of cheese (*15*, *17*).

**Table 6:** ACE-I inhibition by añejo cheese soluble extracts and significant difference regarding ripening time (p≤0.05)

*t*(ripening)/day	ACE-I inhibition/%
0	(6.5±2.0)^c^
30	(23.3±2.0)^b^
95	(23.2±2.0)^b^
180	(39.38±2.0)^a^

Mean value±standard error of six independent determinations corresponding to three independent replicates for each cheese (A and B). Different letters indicate significant differences between ripening times (p≤0.05)

The IC_50_ determined for cheese samples after 180 days of ripening were (0.42±0.04) and (0.38±0.02) mg/mL for A and B cheeses, respectively (data not shown). There are IC_50_ reports for cheeses such as Parmigiano Reggiano (0.017 mg/mL), Grana Padano (0.01 mg/mL) and Mexican fresh (0.005 and 0.011 mg/mL) (*18*, *35*). It is difficult to compare ACE-I inhibition results from different studies due to differences in the inhibition assays and extraction methods used. The reported IC_50_ values have different calculation bases, using total protein content, total peptide content or amount of cheese (*48*). However, the IC_50_ value still gives an idea of the protein concentration required to inhibit 50 % of ACE-I activity using Zacazonapan añejo cheese extracts (*17*).

It should be noted that peptides with antihypertensive activity contain hydrophobic amino acid residues such as Pro, Phe and Tyr at their three C-terminal positions and branched aliphatic amino acids at the N-terminus, as well as positively charged amino acids such as Lys or Arg at the C-terminus (*49*, *50*). In the present study, amino acids related to this bioactivity such as Trp, Phe, Tyr and Arg were identified in the extracts (Table 2). Based on the obtained results, the highest antihypertensive activity was observed in the extracts of cheese B, whose raw materials and processes are different from those of cheese A. Therefore, it is important to clarify the effect of the raw material sources (milk) and processes on the biological activity of the peptides present.

## CONCLUSIONS

The soluble extracts of añejo cheese from Zacazonapan in Mexico showed differences in composition related to the different dairy and ripening time. The free amino acid content increased during ripening due to the gradual enzymatic hydrolysis activity of the casein. The amino acid and peptide profiles for each ripening time could be used to differentiate Zacazonapan añejo cheese from other traditional Mexican cheeses. The soluble cheese extracts contained amino acids associated with bioactivities such as antioxidant and antihypertensive effects. Further studies are needed to identify the molecular mass fractions with greater biological activity, the amino acid sequences of the peptides present and their relationship with bioactivity. These results open up lines of research that will help to preserve the richness of genuine artisanal cheeses, which contribute to their typicality, originality, distinctiveness and uniqueness on the market compared to commercial cheeses.
